# Access to and use of preventive intermittent treatment for Malaria during pregnancy: A qualitative study in the Chókwè district, Southern Mozambique

**DOI:** 10.1371/journal.pone.0203740

**Published:** 2019-01-24

**Authors:** Paulo Arnaldo, Maria Isabel Cambe, Amílcar Magaço, Sérgio Chicumbe, Eduard Rovira-Vallbona, Anna Rosanas-Urgell, Sónia M. Enosse

**Affiliations:** 1 Plataforma de Parasitologia Molecular, Instituto Nacional de Saúde, Maputo, Mozambique; 2 Department of Biomedical Sciences, Institute of Tropical Medicine Antwerp, Antwerp, Belgium; 3 Department of Biomedical Sciences, University of Antwerp, Antwerp, Belgium; 4 Programa de Sistemas de Saúde, Instituto Nacional de Saúde, Maputo, Mozambique; Children's Mercy Hospitals and Clinics Department of Pathology and Laboratory Medicine, UNITED STATES

## Abstract

**Background:**

Malaria remains a significant health problem in Mozambique, particularly in the case of pregnant women and children less than five years old. Intermittent preventive treatment with sulfadoxine-pyrimethamine (IPT-SP) is recommended for preventing malaria in pregnancy (MiP). Despite the widespread use and cost-effectiveness of IPTp-SP, coverage remains low. In this study, we explored factors limiting access to and use of IPTp-SP in a rural part of Mozambique.

**Methods and findings:**

We performed a qualitative study using semi-structured interviews to collect data from 46 pregnant women and four health workers in Chókwè, a rural area of southern Mozambique. Data were transcribed, translated where appropriate, manually coded, and the content analyzed according to key themes. The women interviewed were not aware of the risks of MiP or the benefits of its prevention. Delays in accessing antenatal care, irregular attendance of visits, and insufficient time for proper antenatal care counselling by health workers were driving factors for inadequate IPTp delivery.

**Conclusions:**

Pregnant women face substantial barriers in terms of optimal IPTp-SP uptake. Health system barriers and poor awareness of the risks and consequences of MiP and of the measures available for its prevention were identified as the main factors influencing access to and use of IPTp-SP. Implementation of MiP prevention strategies must be improved through intensive community health education and increased access to other sources of information. Better communication between health workers and ANC clients and better knowledge of national ANC and IPTp policies are important.

## Introduction

Pregnant women are at high risk of malaria and its adverse consequences. An estimated 55 million women become pregnant each year in malaria-endemic areas, and of these approximately 10,000 die of malaria [1,2]. Malaria infections during pregnancy are often asymptomatic, but can still cause adverse effects for both the mother and her child, including maternal anemia, impaired fetal growth, premature and still-birth, low birth weight, and congenital malaria, which have been associated with a high risk for infant mortality and morbidity [1,3].

The control and prevention of malaria in pregnancy (MiP) is an important strategy to avert malaria adverse consequences and improve maternal and infant health. For areas with moderate and high malaria transmission in Africa the World Health Organization (WHO) recommends a package consisting of intermittent preventive treatment in pregnancy (IPTp) with sulfadoxine-pyrimethamine (SP), use of insecticide-treated bed nets, and effective case management of clinical malaria and anemia [4,5]. IPTp-SP and bed nets are delivered during antenatal care (ANC) visits. Although many countries in sub-Saharan Africa (SSA) have adopted IPTp-SP for MiP,coverage of the recommended IPTp-SP dosage (≥ 3 doses) is still unacceptably low among pregnant women, despite a modest increase in ANC attendance [3], This low coverage limits the beneficial effect of IPTp-SP on maternal and child outcomes [6,7].

Quantitative data collection approaches have been widely used to explore factors affecting access and use of IPTp-SP. Factors associated with low coverage include limited access to ANC services, attitudes and practices among health care professionals, low awareness of the consequences of MiP, poor patient adherence, and community attitudes towards preventive interventions [8–11]. Quantitative studies, however, often do not capture sociocultural aspects such as sociocultural beliefs among individuals and community that may be affecting pregnant women’s access to and use of malaria control interventions [10,12–14].

Countrywide policies aimed at improving maternal and neonatal health have been implemented in Mozambique. Specifically, these policies target anemia and malnutrition, prevention of MiP, increased rates of institutional deliveries, delayed age of first pregnancy, and reduced practice of unsafe abortions [15,16]. IPTp-SP has been administered in Mozambique using the directly observed therapy approach since 2006, and insecticide-treated bed nets are distributed free of charge to pregnant women at ANC visits [15]. Also, national guidelines were recently updated to ensure the countrywide implementation of the WHO recommendation of administering at least 3 doses of IPTp-SP to pregnant women [16]. According to data from the 2015 national survey 34.2% of pregnant women in Mozambique were receiving two doses of IPTp-SP while 22.4% were receiving three [17]. Data, however, from a recent study by our group in the rural Chókwè district showed a coverage rate of 46.6% for three or more IPTp-SP doses at the time of delivery [18].

The current study explored perceptions, experiences, and behaviours among key informants (pregnant women and health workers) regarding malaria prevention in pregnancy (IPTp-SP) in order to identify individual and sociocultural factors that influence uptake of IPTp-SP during ANC visits. Finding from this study will provide information which could assist malaria control programs to implement appropriate strategies to improve the overall IPTp-SP coverage in the region and in the country.

## Materials and methods

### Study site and population

The study was conducted in the health and demographic surveillance system (HDSS) catchment area of the Chókwè district, Gaza Province, Mozambique. Chókwè is a rural district is situated on the Limpopo River and most of its population belongs to the Changana ethnic group, whose main economic activities are subsistence farming, large-scale rice production, livestock keeping, small business and migrant labour in South Africa. Around 135,000 habitants are under continuous follow-up through the HDSS. This system covers an area of approximately 600Km^2^ within a 25Km radius of Chókwè City. The HDSS is run by the “Centro de Investigação e Treino em Saúde de Chókwè” (CITSC), a clinical research center affiliated with the Instituto Nacional de Saúde, which is overseen by the country’s Ministry of Health. The HDSS routinely registers pregnancies, births, deaths, and migrations [Bonzela et al, in preparation]. There are two seasons: a hot, rainy season that runs from November to April and a cool, dry season that runs from May to October. Malaria transmission is perennial and occurs year-round, although it is more intense during the rainy season. *Plasmodium falciparum* is the predominant malaria parasite species in the area [19].

At the time of data collection, the country had adopted the new WHO policy recommendation that calls for monthly SP administration and a minimum of three doses during the course of pregnancy [20]. Within the HDSS catchment area the official health Network is comprised by nine health centres. The referral district hospital is Chókwè Rural hospital with 125 beds and the Carmelo hospital which is specialized in TB and HIV management. Most of the government medical services are provided free of charge except for drugs prescribed at the outpatient department, which are available for purchase at subsidized prices. The other seven health centres provide maternal and child health care and preventive services, screening and treatment of syphilis, anemia, and urinary tract infections, administration of anthelmintic treatments, ferrous sulphate supplementation, folate tablets, and tetanus toxoid vaccines, and prevention of mother to child transmission of HIV [15]. The prevalence of HIV in Mozambican women aged 15–49 years in 2015 was 28.2% in 2015 [17].

### Study design and data collection

This was a descriptive qualitative study undertaken between March and April 2015 in the context of a study conducted in the same area designed to evaluate IPTp-SP uptake and pregnancy outcomes in order to explore barriers to IPTp for preventing MiP. Four primary health facilities were selected for data collection. To qualify for participation in the study, the health center had to be located in the study area and offer maternal and child health care and preventive services. Therefore, the Chókwè Health Center, Terceiro Bairro Health Center, Lionde Health Center and Conhane Health Center were selected into the study.

At each of the four facilities, interviews were held with a sample of health service users, represented by pregnant women aged ≥15 years old, and health workers, represented by nurses. Pregnant women were randomly selected from those who visited the health facilities for prenatal consultations and provided their written informed consent to participate in the study. One nurse was selected at each of the health facilities. To qualify for participation in the study, the nurse had to have been delivering ANC for at least 1 year before the interview.

Interviews were held in a private room at the healthy facility and conducted by an experienced male social scientist assisted by a female research officer specifically trained for this study. Sessions ran for approximately 45 minutes and were conducted in Portuguese and/or in Changana (local language) depending on the participants’ preferences. All interviews were digitally recorded.

Interview guides were developed to explore factors limiting access to IPTp during pregnancy from the perspectives of both the pregnant women and the nurses. The Pregnant women were interviewed using a semi-structured questionnaire focusing on (*a*) general perception of diseases in the study area, (*b*) perceptions of malaria and IPTp-SP, and (*c*) experiences with ANC and perceptions of the quality of service (S1 Form). The nurses were asked about (*d*) women’s attitudes towards IPTp and challenges for IPTp-SP delivery (S2 Form).

### Transcription and data analysis

The full content of the interview recordings in the local language (Changana) was transcribed *verbatim* and translated into Portuguese. All transcripts were read for accuracy before the analysis. Data were coded separately according to the original research questions and the data collection guides. They were coded using pre-defined themes based on the research questions and analyzed manually using a content data analysis method, which involved familiarization with data through reading and re-reading of transcripts and refining of themes by comparing codes with research questions. The headings used in the results and discussion sections of this paper reflect the codes used for the analysis.

### Ethical approval and consent to participate

The study was approved by the National Health Bioethics Committee (CNBS) (IRB 00002657). Administrative approval to conduct the study was obtained from the local health facilities and the Ministry of Health of Mozambique. With participants' prior agreement, written informed consent was obtained prior to the interview. Women under 18 years of age provided informed assent and their husbands, mothers, or representatives provided informed consent. During transcription, names were replaced with codes to ensure anonymity and digital recordings were deleted once the transcription and translation had been completed and checked for quality.

## Results

Fifty participants—46 pregnant women and four health workers (nurses)—were recruited and interviewed. The demographics of the pregnant women are provided in Table 1.

**Table 1:** Characteristics of pregnant women included in the study (N = 46).

Characteristic	Frequency (%)
Median age [IQR] (years)	25.5 [22–32]
*Education level*	
No formal education	7 (15.5)
Primary	22 (48.8)
Secondary or higher	17 (37.7)
*Marital Status*	
Married	6 (13.3)
Single	17 (37.7)
Marital union	23 (51.1)

All the nurses had been trained in maternal and child health care and prevention. Their level of education ranged from secondary to higher. Two of the nurses had been working in Maternal and Child Health Services for at least 3 years and the other two had been working for 1 year. Predefined themes were coded around the four key topics in the topic guide: (*a*) general perceptions among pregnant women of diseases in the study area; (*b*) perceptions among pregnant women of malaria and IPTp-SP; (*c*) experience with ANC services and perceptions among pregnant women of service quality; (*d*) perceptions among nurses of challenges for IPTp-SP delivery (S1 Table).

### *a*. General perceptions of diseases among pregnant women

When responding to the open-ended question about perceived illnesses affecting the population, the pregnant women identified HIV/AIDS, malaria, tuberculosis and diarrhoea as the most common health problems in the area. Other diseases cited as less prevalent but that affected pregnant women were *Xibelekelo* (native language)—described as a disease linked to the female reproductive system (specifically to the uterus) that causes intense pain and affects women during the cold season—and *xitsongua tsonguana*—described as a disease that affects pregnant women characterized by convulsions and high blood pressure. According to local belief *Xitsongua tsonguana* can be passed from the mother to the baby, and when this happens, the baby can be born with "moon disease" which refers to a set of symptoms including body pain, convulsions, fevers, constipation, and cough that the baby may experience each time a full moon appears. Other diseases cited by participants were *Dzedzedze* (fever), stress, and diabetes.

### *b*. Perceptions of malaria and IPTp-SP among pregnant women

The majority of pregnant women (80.4%, 37/46) perceived malaria as one of the main diseases in the local population and mentioned that it mainly occurred in the hot, rainy season. The main symptoms mentioned were hot body, headache, vomiting, weakness, lack of appetite, body pain, and joint pain. Quinine and Coartem were mentioned as the most common antimalarial drugs available.

“*I know that muntzototo (malaria) is the main health problem during rainy season*. *I got muntzototo (malaria) more than once*, *but I always got treated*.*”* [pregnant woman].

Pregnant women were unanimous in characterizing malaria as a disease widely known to attack everybody, and they perceived mosquito bites as the main cause of infection. A dirty environment with stagnant water was also mentioned as an important cause of malaria infection as it can increase mosquito population. The study participants correctly observed that malaria infection can cause anemia and death, and perceived pregnant women and children as the groups most at risk for malaria infection and adverse consequences. However, when asked about the specific deleterious effects of MiP, very few (10.9%, 5/46) were able to mention these.

*“Pregnant women are the people who can contract muntzototo (malaria) very easily*, *I do not know how to explain this*, *but this is the period of conceiving another life”-* [pregnant woman].

Most of the women (89.1%) were aware that malaria is preventable and recognized that the importance of keeping the house clean and using mosquito nets. Their beliefs about malaria prevention encompass hygiene, a clean environment, and use of mosquito nets, but they did mention the use of medicines such as tablets.

*“We have to clean up where we live*, *especially when sleeping*, *windows must be closed and people should sleep under a mosquito net"-* [pregnant woman].

More than half women (56.5%, 26/46) mentioned having heard about drugs given at the ANC clinic to prevent diseases in general during pregnancy, but most of them were unable to mention either the names of the drugs or the reasons they were given. They mentioned having received "*comprimidos*" (Portuguese word for tablets) to refer to the three drugs given to pregnant women during ANC malaria prevention visits, although very few were able to specify which tablet. Just 10.8% named it as *Fansidar*, a commercial name for SP. In order to further explore the topic of IPTp, the interviewer referred to SP as ''the white tablets given to pregnant women and normally taken in front of the nurse''.

*"I did not know the reason I was given these tablets*. *They only told me to take three tablets*, *and then gave me some more "comprimidos vermelhos"(red tablets)*, *which refers to iron supplement tablets”-* [pregnant woman].

When the women who mentioned that they had received SP tablets in context of IPTp were asked why they had taken the treatment, some of them said that they had started it because they were sick, that they did not want their baby to be born sick, and that they wanted to prevent the baby from getting other diseases. However, they had no detailed knowledge about the recommended dosage.

“*I went early to my first antenatal care visit to know if I had any diseases*, *to get checked*, *and receive drugs for malaria prevention in order to protect my baby*. *We cannot allow many months to pass without preventing malaria because if you do this when you are already sick the child can suffer and the doctors cannot do anything*.*”* [pregnant woman]

### *c*. Experiences with ANC services and perceptions of service quality among pregnant women

Experiences with ANC visits and perceptions of quality were positive. Those interviewed largely said that they had good interactions with the staff at the health care facility, were given information about malaria and how to prevent it, and were offered HIV testing. There were, however, also some negative experiences. Some women mentioned that information about malaria and other important health issues should be given not only through counseling during visits to the clinic but also during daily lectures at the start of working days held at the health centers. Others mentioned having been given tablets but without any explanation.

***“****They said we have to come to the hospital for antenatal care and for counselling*. *I registered and was tested for HIV and the result was negative*. *The nurse gave me all the information and offered me tablets*. *She also provided me ferrous salt and told me to take it once a day*, *I also received a mosquito net”-* [pregnant woman].

*“Were you given any explanation about the benefits of taking those tablets*?*”* [interviewer]

*“No*, *I was only given the tablets and I accepted*. *They did not explain anything to me*, *they just gave them and said you have to swallow them here*.*”-* [pregnant woman]

The women also complained of long waiting times and mentioned that these delays influenced their uptake of ANC services. This was a particularly important factor for patients living far from the health facility, especially if they had other children under their care. One pregnant woman who had her first ANC visit at six months of gestation, said:

*" This [waiting time] makes it a bit difficult because I live far away from the health center when I imagine that I will stay here for a long time I give up"-* [pregnant woman].

### *d*. Perceptions of challenges for IPTp-SP delivery among nurses

Although (76.1%, 35/46) of pregnant women interviewed mentioned having received three white tablets at least once during ANC visits, the nurses claimed that women do not receive the recommended three or more doses of IPTp-SP during the gestational period. This was explained by the nurses in part by the fact that pregnant women delay seeking care and attend antenatal clinics for the first time during the third trimester (at 7–8 months of gestation), thereby greatly reducing the chances of receiving three or more IPTp-SP as recommended.

"*Women come for the first ANC visit at thirteen weeks of pregnancy and may receive up to three doses*, *but in most of the cases*, *women books first visit at eight months*. *These will not complete the recommended doses* "-[maternal and child nurse].

The main barrier to the effective delivery of health services such as IPTp-SP in the opinion of the nurses interviewed was the high patient load. The nurses recognized that in most cases SP tablets are given to pregnant women without any explanation about their purpose or benefits because the staff are overloaded with patients and are “forced” to perform quick consultations in order to manage the pressure they are under.

*" Usually*, *we are overworked*, *with much to do*. . . . *And when the women come for a prenatal consultation*, *I often just give the tablets for malaria prevention*, *sometimes even without explaining carefully the details "-* [maternal and child nurse].

*"The person [pregnant women] says that [*…*] I cannot come to the hospital soon because it gets very crowded*, *so I prefer to come here just for delivery"-* [maternal and child nurse].

The nurses also mentioned that IPTp-SP uptake was influenced by the lack of educational materials such as pamphlets, pictures or figures on MiP and its consequences as this made it difficult for them to inform future mothers on the beneficial effect of this treatment.

*“It is difficult to notice that pregnant women who are at higher risk who do not seek treatment or do not take the completed IPTp dosage*. *Perhaps if we had some illustrative pictures or images of a person who did not take complete treatment*, *it would be helpful*. *I think that this is a bit of a perception because some do not complete the recommended dosage because they think the tablets are strong*, *in this way even with our counselling*, *women will not complete the dosage”*.*-* [maternal and child nurse].

## Discussion

This qualitative study investigating perceptions and opinions of key informant groups (pregnant women and health workers) in the Chókwè district provides insights that should prove valuable for informing strategies within the national malaria control program aimed at improving IPTp-SP delivery and uptake. Our findings support quantitative findings from the study that was undertaken in parallel to this study with regard to IPTp-SP coverage among pregnant women at delivery that reported similarly low levels of awareness and knowledge of IPTp for malaria prevention in the same area [18].

Although women perceived malaria as one of the main diseases affecting the local population, they did not appear to be aware of the risk and specific deleterious effects posed by MiP to both them and the fetus. This observation is consistent with reports from another study conducted in Manhiça district, southern Mozambique that pregnant women were not fully aware of adverse maternal and birth outcomes associated with malaria [10]. The low awareness of malaria risk and its adverse consequences during pregnancy observed in this study might negatively influence adherence to IPTp-SP and general malaria prevention interventions. It also suggests the need for better strategies at ANC clinics and in the community to increase awareness about the risk and consequences of MiP and the benefits of SP and other MiP prevention strategies [21, 22]. In this respect, however, a closer analysis of the contents and format of the health education sessions and other sources of information in the community is necessary.

Women recognized the importance of keeping their homes clean and using mosquito nets as a means of preventing malaria. However, they failed to fully recognize the value of medicines/tablets, such as IPTp-SP for MiP prevention, supporting findings from Kenya [23]. The results of the present study can be considered robust, as women failed to mention the main strategy for MiP prevention (IPTp-SP) not only when discussing malaria prevention approaches in general, but also were probed about SP in particular. Similar findings were reported in a systematic review of factors affecting access to and delivery and use of MiP prevention strategies in sub-Saharan Africa, in which the majority of women were found to be unaware of the use and benefits of IPTp-SP for this purpose [21]. Health services specifically targeting pregnant women and strategies to encourage them to attend ANC visits would appear to be essential. In addition, health promotion programmers need to ensure that women and other community members are provided with accurate information on MiP prevention measures to reinforce attitudes on IPTp-SP uptake.

Although most of the pregnant women interviewed had not heard of IPTp-SP for malaria prevention, they did remember that they had been given a drug during ANC visits. While some of them could not remember the name of the drug or were not aware that it was for preventing malaria, they were able to describe the three white tablets they had taken in front of the nurse. Previous studies in Africa have found perceptions of adverse reactions to drugs to be important determinant of treatment adherence [10,11,24]. In this study, there were no indications that perceived adverse reactions had a negative influence on adherence, since none of the respondents expressed concerns over the safety of IPTp-SP.

The women interviewed recognized that health workers were “well-intentioned” during ANC visits, although they did mention some negative aspects that could be barriers to IPTp-SP delivery. Particular mention was made of the practice of giving SP tablets to pregnant women without an explanation about what the tablets were for. Other important factors mentioned by the pregnant women and nurses as potential barriers to the use of ANC services and uptake of IPTp-SP were long waiting times and absence of supporting materials such as illustrative pictures or images and pamphlets to use during health education sessions. These observations are consistent with reports from many settings in sub-Saharian Africa [11,23,25–27] and add to the growing body of evidence calling for improvements in the quality of ANC in the study area and in Mozambique in general.

Individual barriers to adequate IPTp-SP uptake mentioned by the nurses interviewed were late and irregular ANC attendance. The impact of late and irregular attendance to ANC visits by pregnant women has been reported elsewhere [28]. Similarly, studies in Uganda and Mali also found that late and irregular ANC attendance were important factors influencing poor uptake of IPTp-SP and other malaria prevention measures [29,30]. A better understanding of the factors that deter early ANC-seeking behaviors during pregnancy is essential for improving IPTp-SP coverage. Some studies have reported a link between late and irregular ANC attendance and cultural beliefs among pregnant women, including, for example, a reluctance to disclose their pregnancy in the early stages, particularly among adolescents, the tendency to only start attending ANC visits when the belly is visible (generally after month 5), and a tendency to only seek help when sick [14,29,30].

Missed opportunities have also been shown to be important barriers to adequate IPTp uptake [7]. Although data show that the vast majority of pregnant women in the Gaza province of Mozambique attend four ANC visits [17], the particularly low attendance reported by health workers in the Chókwè district in this study is consistent with reports from the same area [18]. It should be noted, however, that since no SP stockouts have been observed in the Chókwè district, it is possible that women were not always supplied with IPTp-SP during their ANC visits, as has been previously reported [8,30].

Challenges for effective IPTp-SP delivery mentioned by the nurses interviewed included the high number of patients and workloads. This “overload” offers some explanation as to why women are not always given detailed information about MiP and IPTp strategies during the ANC visits. This lack of information is likely to be associated with poor uptake of MiP prevention measures in general [31]. Our observations in this respect suggest that adequate deployment of health staff is needed to counteract the high workload mentioned and to ensure the quality of health services provided.

Our study findings should be interpreted in light of several limitations: First, the interview with the pregnant women were held at the health facility attended by the women. Some of the women therefore may not have felt entirely comfortable with openly giving their opinions on the questions raised by the interviewer. The selection of participants also meant that we missed out on insights from pregnant women in the community who do not attend ANC visits. Finally, we did not collect data through focal group discussions, which would have enabled us to explore in greater depth experiences and beliefs concerning access to and use of IPTp-SP access among different population strata in the community. Future research, however, could explore further dimensions of factors influencing the delivery of preventive interventions for MiP.

## Conclusions

This study has shown that access to and use of IPTp-SP in the rural Chókwè district of Mozambique are affected by various social factors, but in particular by poor awareness of the risks and consequences of MiP and the specific benefits of IPTp-SP and delayed ANC attendance. Health system barriers such as long wait times and high patient loads resulting in insufficient time for proper counseling were also shown to negatively influence IPTp-SP uptake Intensive health education within the community and other strategies to increase awareness among women are necessary to improve the delivery of MiP prevention interventions. It is also important to ensure better communication between health service providers and ANC clients and to promote a better understanding of national ANC and IPTp policies.

## Supporting information

S1 Form Interview guide for pregnant women. (PDF)

S2 Form Interview guide for health workers. (PDF)

S1 Table Factors limiting the access to IPTp intervention for malaria prevention in pregnancy. (PDF)
