# Supplementary material for: Access to and use of preventive intermittent treatment for Malaria during pregnancy: A qualitative study in the Chókwè district, Southern Mozambique
Source: PLoS One. 2019 Jan 24;14(1):e0203740. doi: 10.1371/journal.pone.0203740 (PMC6345468; doi:10.1371/journal.pone.0203740)
Supplement: S1 Form — (PDF) [file pone.0203740.s001.pdf]

S1 Form Interview guide with Pregnant women

(a) General perception of diseases affecting the population in the study area among pregnant women

What are the most common diseases in pregnant women and children in your community?

a) What are the most frequent illnesses in raining season?
b) What are the most frequent diseases in cold season?
c) What should a pregnant woman do to protect herself from getting diseases?

b) Perceptions of malaria and IPTp-SP among pregnant women

What is your knowledge about malaria?

→ Explore the following aspects:

- Transmission and prevention methods
  - If there is no mosquitoes can you still get malaria?
- Main symptoms and treatment
  - How does a person perceive that they have malaria?
- Causes and consequences/effects
  - What is you perception about the risk of getting malaria during pregnancy?
  - Do you think pregnant women are more vulnerable than others to get malaria?, Why?
  - How pregnant women feel when she has malaria, what can happen to her and to the baby?

What are the malaria prevention methods you know?

Probe:

- What malaria prevention methods have you been using?

- Did hear about any medicines that are used to prevent malaria during pregnancy?

If YES → Which one ? (name it), What color were the medicines?

- Where you had that information?
- Have you ever received antimalarial tablets during your pregnancy?
- If yes , where and how many tablets ?
- What are the advantages can this medication bring to the pregnant women?
- Did you notice any side effect after taking the tablets?
- IF YOU DID NOT TAKE ... → Why did you never take it?

***c)* Experiences with ANC services and perceptions of service quality among pregnant women**

- What motivated you to came for antenatal consultation?
- Do you think you came early or late to this antenatal consultation
- How many time did you come for antenatal consultation?
- What did the nurse do to you during this consultation?
- Do you remember about anything about malaria that you have told during the consultation?

  If yes. → Can you tell what they told you?
- Did you receive any medication?
- How many tables you received?
- Did the nurse explain to you the propose of taking that medicines?

  → If no, Why you didn't consult?

Have you talked to the nurse during the consultation?

- What have you talked about with her?
- If No , Why you never talk to the nurse?
- In general, what is your opinion about the qualities of the services provided to you as pregnant women in the antenatal consultations in your health unit?
- Would you like to say something more about the ANC services in this health center?
