# Supplementary material for: Access to and use of preventive intermittent treatment for Malaria during pregnancy: A qualitative study in the Chókwè district, Southern Mozambique
Source: PLoS One. 2019 Jan 24;14(1):e0203740. doi: 10.1371/journal.pone.0203740 (PMC6345468; doi:10.1371/journal.pone.0203740)
Supplement: S2 Form — (PDF) [file pone.0203740.s002.pdf]

S2 Form Interview guide with Nurses of Maternal Child Health

Perceptions of pregnant women's attitudes towards IPTp-SP use among nurses

A1. On average how many pregnant women do you attend per day?

a) In which gestational age the women normally appear for the first ANC consultation?
b) How many ANC visits they normally do?

A2. Are there any criteria for administering antimalarial drug to prevent malaria in pregnant women?

A3. At what gestational age do you recommend them start taking antimalarial drug to prevent malaria??

A4. How frequent are malaria cases in pregnant women?

A5. Which period you have seen the highest number of malaria tested women?

a) Of those tested on a given day, how many of them have the positive diagnosis?

b) What procedures do follow when a woman has tested positive?

A6. Are antimalarial drugs administered at the clinic or the women has to pick the up at the pharmacy?

a) How long does the process of the consultation and antimalarial administration usually last?

A7. Is there any explanation given to pregnant women during drug administration?

→ If yes, what do they explain exactly?

→ If not, why not?

A8. What do you think about pregnant women knowledge about importance of using antimalarial drugs during pregnancy?

a And how do you come to that perception?

b) Has any pregnant women ever refused to take this antimalarial drugs?

→ If yes, what are the reasons?

A9. In general, how many pregnant women receive the recommended dosage of IPTp?

Perceptions of challenges for IPTp-SP delivery among nurses

A10. What are the difficulties you are facing to get pregnant women comply with the recommended dosage of IPTp?

→ Difficulties of the system;

→ Difficulties of the behavior of the providers; Difficulty of patient behavior.

(Detail)

A12. Have you ever had some antimalarial stockouts?

A13. What is your perception about community perception about malaria prevention in this area
