# Supplementary material for: Access to and use of preventive intermittent treatment for Malaria during pregnancy: A qualitative study in the Chókwè district, Southern Mozambique
Source: PLoS One. 2019 Jan 24;14(1):e0203740. doi: 10.1371/journal.pone.0203740 (PMC6345468; doi:10.1371/journal.pone.0203740)
Supplement: S1 Table — (PDF) [file pone.0203740.s003.pdf]

S1 Table: Factors limiting the access to IPTp intervention for malaria prevention in pregnancy

**Themes**	**Sub-themes and quotations from study participants**
**General perceptions about diseases**	
**Perceptions of malaria and IPTp-SP**	**To know about malaria as important diseases affecting populations.**<br>*"I know about muntzototo (malaria) is the main health problem during rainy season. I got muntzototo (malaria) more than once, but I always got treated"*[pregnant woman]
	**Malaria risk groups and its adverse consequences to pregnant women and children**<br>*"Pregnant women are the people who can contract muntzototo (malaria) very easily, I do not know how to explain this, but this is the period of conceiving another life."* [pregnant woman]
	**To test knowledge about malaria prevention**<br><br>*"We have to clean up where we live, especially when sleeping, windows must be closed and people should sleep under a mosquito net*." [pregnant woman]
	**To test on Pregnant women's perceptions about IPTp**<br><br>*"I did not know the reason I was given these tablets. They only told me to take three tablets, and then gave me some more "comprimidos vermelho"(red tablets), which refers to iron supplement tablets"* [pregnant woman]<br><br>*"I went early to my first antenatal care visit to know if I had any diseases, to get checked, and receive drugs for malaria prevention in order to protect my baby. We cannot allow many months to pass without preventing malaria because if you do this when you are already sick the child can suffer and the doctors cannot do anything."* [pregnant woman]
**Pregnant women's experiences and perceptions of ANC service quality**	**To test on Pregnant women's perceptions about ANC service quality**<br><br>**"***They said we have to come to the hospital for antenatal care and for counseling. I registered and was tested for HIV and the result was negative. The nurse gave me all the information and offered me tablets. She also provided me ferrous salt and told me to take it once a day, I also received a mosquito net.*" pregnant woman]<br><br>*"Were you given any explanation about the benefits of taking those tablets?"* [interviewer]

	“No, I was only given the tablets and I accepted. They did not explain anything to me, they just gave them and said you have to swallow them here.” [pregnant woman]<br><br>*" This [waiting time] makes it a bit difficult because I live far away from the health center when I imagine that I will stay here for a long time I give up*"- [pregnant woman]
**Health providers perceptions of challenges for IPTp-SP delivery**	*" Women come for the first ANC visit at thirteen weeks of pregnancy and may receive up to three doses, but in most cases, women book their first visit at eight months. These will not complete the recommended doses."* [maternal and child nurse]<br><br>*"Usually, we are overloaded, with much to do.... And when the women come for a prenatal consultation, I often just give the tablets for malaria prevention, sometimes even without explaining carefully the details."* [maternal and child nurse]<br><br>*"The person [pregnant women] says that [...] I cannot come to the hospital soon because it gets very crowded, so I prefer to come here just for delivery."* [maternal and child nurse]<br><br>*“It is difficult to notice pregnant women who are at higher risk who do not seek treatment or do not take the completed IPTp dosage. Perhaps if we had some illustrative pictures or images of a person who did not take the complete treatment, it would be helpful. I think that this is a bit of a perception because some do not complete the recommended dosage because they think the tablets are strong, in this way even with our counseling, women will not complete the dosage.”* [maternal and child nurse]
